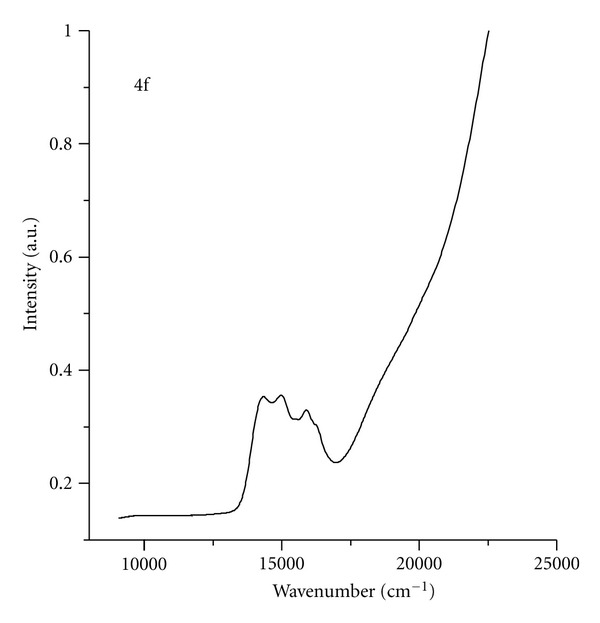# Synthesis of Furo[3,2-*b*]pyrrole-5-carboxhydrazides and Their Cu, CO and Ni Complexes

**DOI:** 10.1100/2012/915798

**Published:** 2012-04-19

**Authors:** Renata Gašparová, Ján Titiš, Filip Kraic

**Affiliations:** Department of Chemistry, University of Ss. Cyril and Methodius, Nám. J. Herdu 2, 91701 Trnava, Slovakia

## Abstract

Carboxhydrazides **3** were synthesized by reaction of substituted furo[3,2-*b*]pyrrole-5-carboxhydrazides **1** with 4-oxo-4*H*-chromene-2-carboxaldehyde **2** in the presence of 3-methyl-benzenesulfonic acid in ethanol. Carboxhydrazides **3** were used as ligands for synthesis of Cu, Co, and Ni complexes **4**.

## 1. Introduction

Carboxhydrazides and their derivatives represent an interesting class of compounds which exhibit antitumor [1], antimicrobial [2], analgesic, and anti-inflammatory [3] activities. Complexes of carbohydrazides are also known for their biological activity. La(I) and Sm(II) complexes of 6-hydroxy chromone-3-carboxaldehyde benzoyl hydrazone were tested against tumor cell lines including HL-60 and A-549 [4]. Zn(II) complex of 4-oxo-4*H*-chromene-3-carboxaldehyde thiosemicarbazone binds to DNA and possesses a significant antioxidant activity [5].

The present study is a follow-up paper to our previous research dealing with the synthesis and reactions of furo[3,2-*b*]pyrrole system [6, 7] and the study of its biological activity. The effect of *N*′-{[5-(R-phenyl)furan-2-yl]methylene}-2-[3-(trifluoromethyl)phenyl]-4*H*-furo[3,2-*b*]pyrrole-5-carboxhydrazides on inhibition of photosynthetic electron transport in spinach chloroplasts and chlorophyll content in the antialgal suspensions of *Chlorella vulgaris *was investigated [8].

## 2. Experimental

Melting points of products were determined on a Kofler hot plate apparatus and are uncorrected. All solvents were predistilled and dried appropriately prior to use. ^1^H NMR spectra were obtained on a 300 MHz spectrometer VARIAN GEMINI 2000 in CDCl_3_ or DMSO-*d*_6_ with tetramethylsilane as an internal standard. Elemental analyses were measured on EAGER 300 apparatus. Electronic spectra were measured in Nujol mull on Specord 200 (Analytical Jena) in the range of 50,000–9,000 cm^−1^. IR spectra were measured on Shimadzu IRAffinity-1 apparatus in KBr.

### 2.1. Synthesis of Ligands **3a–3d**

A mixture of furo[3,2-*b*]pyrrole-5-carbohydrazide **1 **(10 mmol) and 4-oxochromene-3-carboxaldehyde **2 **(10 mmol) was heated for 1–4 h at 50–60°C in ethanol (20 cm^3^) in the presence of 4-methylbenzenesulfonic acid. The solid products were filtered off, dried, and crystallized from ethanol.

***N*′-[(4-Oxo-4H-chromen-3-yl)methylene]-2,3,4-trimethyl-furo[3,2-b]pyrrole-5-carboxhydrazide (tmfupy) (3a)**

For C_20_H_17_N_3_O_4_ (363.4): Mp 251–255°C; react. time 2.5 h; yield: 81%; ^1^H NMR (CDCl_3_): 10.98 (1H, brs, NH); 8.93 (1H, s, H-2); 8.69 (1H, s, H-9); 8.20 (1H, d, *J* = 2.5 Hz, H-5); 7.95 (1H, d, *J* = 7.2 Hz, H-6); 7.68 (1H, ddd, *J* = 8.1, 7.3, 1.6 Hz, H-7); 7.50 (1H, d, *J* = 8.4 Hz, H-8); 6.57 (1H, s, H-6′); 4.05 (3H, s, CH_3_); 2.36 (3H, s, CH_3_); 2.21 (3H, s, CH_3_). IR (KBr): 3446, 2916, 1624, 1538, 1466, 1429, 1331, 1274, 1130, 1027, 781 cm^−1^.

***N*′-[(4-Oxo-4H-chromen-3-yl)methylene]-4-methyl-[1]benzofuro[3,2-b]pyrrole-5-carboyhydrazide (mebfupy) (3b)**

For C_22_H_15_N_3_O_4_ (385.4): Mp 241–245°C; react. time 1 h; yield: 85%; ^1^H NMR (CDCl_3_): 11.71 (1H, brs, NH); 8.83 (1H, s, H-2); 8.55 (1H, s, H-9); 8.16-7.72 (4H, m, H-2′, H-3′, H-4′, H-5′); 7.36-7.22 (4H, m, H-5, H-6, H-7, H-8); 7.15 (1H, s, H-6′); 4.31 (3H, s, CH_3_). IR (KBr): 3442, 2910, 1644, 1628, 1547, 1465, 1431, 1365, 1273, 1142, 1027, 788 cm^−1^.

***N*′-[(4-Oxo-4H-chromen-3-yl)methylene]-4-benzyl-[1]benzofuro[3,2-b]pyrrole-5-carboxhydrazide (bzbfupy) (3c)**

For C_28_H_19_N_3_O_4_ (461.5): Mp 263–266°C; react. time 4 h; yield: 82%; ^1^H NMR (CDCl_3_): 11.69 (1H, brs, NH); 8.81 (1H, s, H-2); 8.56 (1H, s, H-9); 8.17-7.75 (4H, m, H-2′, H-3′, H-4′, H-5′); 7.39-7.29 (4H, m, H-5, H-6, H-7, H-8); 7.27-7.16 (5H, m, Ph); 7.13 (1H, s, H-6′); 5.65 (2H, s, CH_2_). IR (KBr): 3442, 2909, 1648, 1630, 1541, 1468, 1429, 1357, 1273, 1138, 1026, 776 cm^−1^.

**2-Methyl-*N*′-[(4-oxo-4*H*-chromen-3-yl)methylene]-4*H*-furo[3,2-*b*]pyrrole-5-carboxhydrazide* (mefupy)* (3d)**

For C_18_H_13_N_3_O_4_ (335.3): Mp 253–256°C; react. time 2.5 h; yield 60%; ^1^H NMR (DMSO-*d*_6_): 11.53 (1H, s, NH); 11.46 (1H, brs, NH); 8.84 (1H, s, H-2); 8.19-8.16 (2H, m, H-5, H-9); 7.87 (1H, ddd, *J* = 7.2, 6.9, 1.5 Hz, H-7); 7.75 (1H, d, *J* = 7.8 Hz, H-8); 7.57 (1H, ddd, *J* = 8.1, 6.9, 1.2 Hz, H-6); 7.01 (1H, s, H-6′); 6.27 (1H, s, H-3′). IR (KBr): 3441, 2890, 1640, 1631, 1541, 1471, 1443, 1358, 1273, 1135, 1022, 762 cm^−1^.

### 2.2. Synthesis of Complexes **4a–4g**

Ligand **3** (0.5 mmol) was dissolved in acetone (10 cm^3^) at 70°C. A solution of metal (M^2+^) nitrate or chloride [Ni(NO_3_)_2_·6H_2_O, Co(NO_3_)_2_·6H_2_O, CoCl_2_·6H_2_O, CuCl_2_·2H_2_O] (0.5 mmol) was added dropwise. Precipitate which was formed immediately, was filtered off, washed with acetone (3 × 15 cm^3^), and dried.

**[Ni(tmfupy)NO_3_]NO_3_·H_2_O (4a)**

Yield: 80%; Anal. Calcd. for C_20_H_19_N_5_NiO_11_ (564.1); C, 42.58; H, 3.40; N, 12.42; Ni, 10.41. Found: C, 42.97; H, 3.21; N, 12.57; Ni, 11.69%. IR (KBr): 3446, 2343, 1630, 1616, 1574, 1419, 1375, 1261, 1136, 1018, 754 cm^−1^.

**[Co(mebfupy)NO_3_]NO_3_·H_2_O (4b)**

Yield: 88%; Anal. Calcd. for C_22_H_17_CoN_5_O_11_(586.3); C, 45.07; H, 2.92; N, 11.94; Co 10.05. Found: C, 46.96; H, 2.71; N, 12.07 Co, 11.54%. IR (KBr): 3385, 2360, 1616, 1516, 1382, 1263, 1024, 752 cm^−1^.

**[Co(mefupy)NO_3_]NO_3_·H_2_O (4c)**

Yield: 78%; Anal. Calcd. for C, 40.31; H, 2.82; N, 13.06; Co, 10.99. Found: C, 42.12; H, 2.70; N, 13.33; Co, 11.72%. IR (KBr): 3385, 2343, 1627, 1605, 1558, 1384, 1261, 1141, 1029, 758 cm^−1^.

**[Cu(tmfupy)Cl_2_]H_2_O (4d)**

Yield: 81%; Anal. Calcd. for C_20_H_19_Cl_2_CuN_3_O_5_ (515.8); C, 46.57; H, 3.71; N, 8.15; Cu, 12.32. Found: C, 45.45; H, 3.62; N, 7.79; Cu, 11.95%. IR (KBr): 3414, 2344, 1631, 1616, 1587, 1560, 1419, 1375, 1267, 1141, 1067, 767 cm^−1^.

**[Cu(bzbfupy)Cl_2_]H_2_O (4e)**

Yield: 93%; Anal. Calcd. for C_28_H_21_Cl_2_CuN_3_O_5_ (613.9); C, 54.78; H, 3.45; N, 6.84; Cu, 10.35. Found: C, 52.03; H, 3.02; N, 6.48; Cu, 10.84%. IR (KBr): 3421, 2343, 1630, 1616, 1584, 1419, 1325, 1269, 1155, 1032, 696 cm^−1^.

**[Co(mefupy)Cl_2_]H_2_O (4f)**

Yield: 77%; Anal. Calcd. for C_18_H_15_Cl_2_CoN_3_O_5_ (483.2); C, 44.74; H, 3.13; N, 8.70; Co, 12.20. Found: C, 45.06; H, 3.24; N, 9.30; Cu, 11.45%. IR (KBr): 3385, 2364, 1631, 1616, 1602, 1577, 1431, 1323, 1227, 1136, 1095, 761 cm^−1^.

**[Cu(mefupy)Cl_2_]H_2_O (4g)**

Yield: 84%; Anal. Calcd. for C_18_H_15_Cl_2_CuN_3_O_5_ (487.8); C, 44.32; H, 3.10; N, 8.61; Cu, 13.03. Found: C, 45.16; H, 3.07; N, 9.04; Cu, 12.88%. IR (KBr): 3431, 2331, 1628, 1616, 1597, 1431, 1325, 1230, 1137, 1031, 758 cm^−1^.

## 3. Results and Discussion

**Scheme 1:**
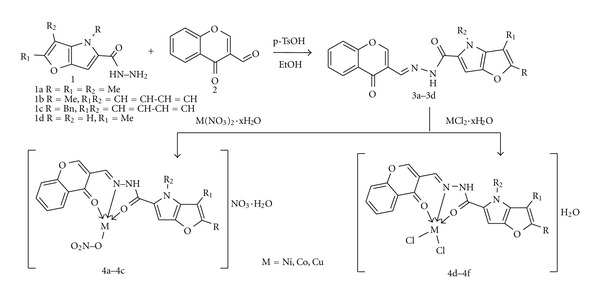
Synthesis of carboxhydrazides **3 **and their complexes **4.**

*N′*-[(4-Oxo-4*H*-chromen-3-yl)methylene]-2-R^1^-3-R^2^-4-R-furo[3,2-*b*]pyrrole-5-carboxhydrazides **3a–3d** were synthesized in 60–85% yields by reaction of **1** with **2** in ethanol in the presence of 4-methylbenzenesulfonic acid by heating at 50–60°C for 1–4 h (Scheme 1).

The ^1^H NMR spectra of compounds **3a–3d** displayed signals of H-2 pyran protons in the 8.81–8.93 ppm range, H-6 pyrrole protons in the 7.36–7.95 ppm range, and signals due to CH=N bonded protons in the 8.19–8.69 ppm range. The chemical shifts and the multiplicity confirmed the proposed structures.

Carboxhydrazides **3 **were subsequently used as ligands in complexation reactions with solutions of metal (M^2+^) chlorides or nitrates [Ni(NO_3_)_2_·6H_2_O, Co(NO_3_)_2_·6H_2_O, CoCl_2_·6H_2_O, CuCl_2_·2H_2_O] in acetone at 70°C. Complexes **4** were obtained in high yields (78–93%).

**Figure 1:**
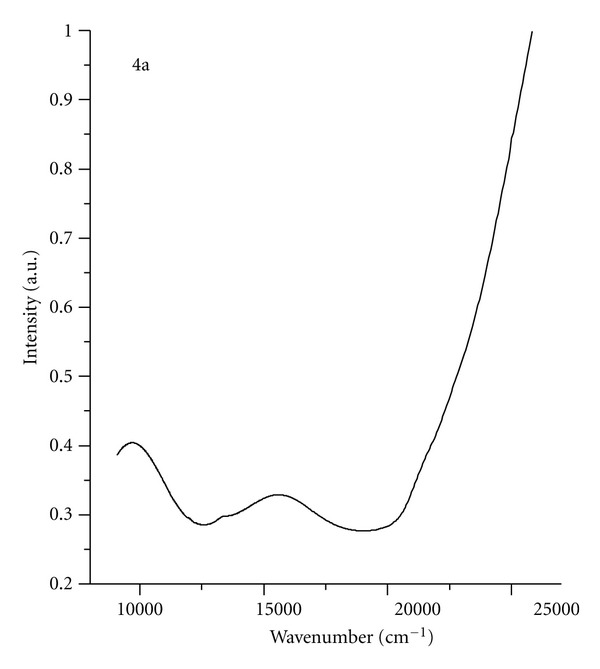


 Structures of **4** were determined by the elemental analyses and electronic spectra. Electronic spectrum of hexacoordinated Ni(II) ion shows transitions at 10000 (^3^A_2g_ → ^3^T_2g_) and 16000 cm^−1^  (^3^A_2g_ → ^3^T_1g_). The third transition (^3^A_2g_ → ^3^T_1g_), which is probably overlapped, should be at about 26000 cm^−1^ (Figure 1).

**Figure 2:**
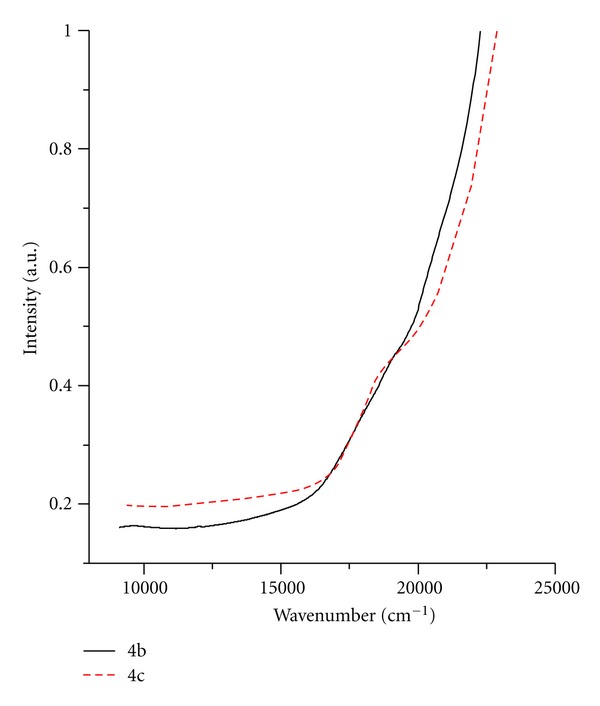


 Electronic spectra of hexacoordinated Co (II) show weak transitions at 15000 cm^−1^  (^4^T_1g_ → ^4^A_2g_) and transitions at 19000 cm^−1^  (^4^T_1g_ → ^4^T_1g_ (P)). Next transitions over 20000 cm^−1^  could be overlapped by strong charge-transfer transitions (Figure 2).

**Figure 3:**
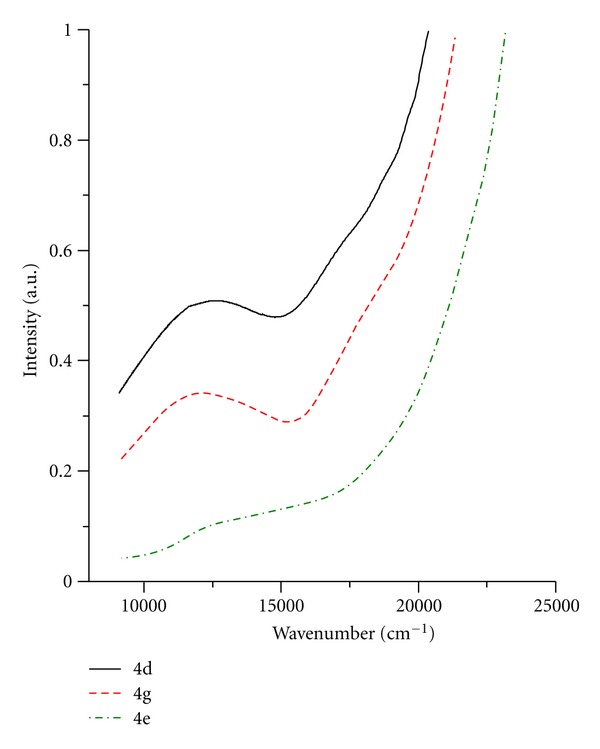


Hexacoordinated Cu(II) ion has tetragonal symmetry with transitions up to 10000 cm^−1^  (^2^B_1g_ → ^2^A_1g_) and 16000 cm^−1^  (^2^B_1g_ → ^2^B_2g_) (Figure 3).

Electronic spectrum of tetracoordinated Co(II) ion shows characteristic transitions in the area of 15000–16000 cm^−1^  (^4^A_2_ → ^4^T_1_(P)) (Figure 4).

## 4. Conclusion

Furo[3,2-*b*]pyrrole-5-carboxhydrazides **1 **reacted substituted with 4-oxo-4*H*-chromene-2-carboxaldehyde **2 **to give *N′*-[(4-oxo-4*H*-chromen-3-yl)methylene]-2-R^1^-3-R^2^-4-R-furo[3,2-*b*]pyrrole-5-carboxhydrazides **3**, which served as ligands for synthesis of Cu, Co, and Ni complexes **4**.

## Figures and Tables

**Figure 4:**